# Towards a new Value-based scenario for the management of dementia in Italy: a SINdem delphi consensus study

**DOI:** 10.1007/s10072-025-08143-5

**Published:** 2025-04-16

**Authors:** Camillo Marra, Flavia Beccia, Paolo Caffarra, Federica L’Abbate, Federica Agosta, Alberto Benussi, Laura Bonanni, Amalia C. Bruni, Giuseppe Bruno, Stefano F. Cappa, Chiara Cerami, Francesco Di Lorenzo, Carlo Ferrarese, Daniela Galimberti, Biancamaria Guarnieri, Claudio Mariani, Benedetta Nacmias, Leonardo Pantoni, Tommaso Piccoli, Daniela Perani, Innocenzo Rainero, Fabrizio Tagliavini, Annalena Venneri, Stefania Boccia, Annachiara Cagnin, Giovanna Elisa Calabrò, Marco Bozzali, On behalf of the SINdem DELPHI Consensus Group, Rossano Angeloni, Gennarina Arabia, Andrea Arighi, Sergio Bagnato, Claudio Babiloni, Roberta Baschi, Giuseppe Bellelli, Valentina Bessi, Edoardo Domenico Giorgio Canu, Sabina Capellari, Moira Ceci, Rosanna Colao, Laura De Togni, Gennaro Della Rocca, Lucia Di Giorgi, Sabrina Esposito, Katia Fabi, Elisabetta Farina, Massimo Filippi, Chiara Fiori, Gianluca Floris, Gianluigi Forloni, Franco Giubilei, Daniela Gragnaniello, Maria Guarino, Alessandro Iavarone, Daniele Imperiale, Valeria Isella, Antonina Luca, Donata Luiselli, Simona Luzzi, Susanna Malagù, Michela Marcon, Alessandro Marti, Alessandro Martorana, Maria Giuseppina Mascia, Patrizia Mecocci, Daniele Mei, Paola Merlo, Fabio Moda, Roberto Monastero, Giuseppe Mura, Anna Maria Musso, Anna Vittoria Marta Orsini, Piero Parchi, Matteo Pardini, Lucilla Parnetti, Andrea Plutino, Gianfranco Puccio, Sabina Roncacci, Mara Rosso, Elisa Rubino, Stefano Sensi, Grazia Sibilia, Marco Spallazzi, Patrizia Sucapane, Pietro Tiraboschi, Gloria Tognoni, Gabriele Tripi, Alessandro Vacca, Marco Vista, Gianluigi Zanusso, Marta Zuffi

**Affiliations:** 1 Department of Psychology, Catholic University of the Sacred Heart, Milan, Italy; 2 Memory Clinic, Fondazione Policlinico Universitario “A. Gemelli” IRCCS, Rome, Italy; 3 Section of Hygiene, University Department of Life Sciences and Public Health, Università Cattolica del Sacro Cuore, Rome, Italy; 4 Membro Tavolo Permanente Demenze- ISS- Ministero della Salute, University of Parma, Parma, Italy; 5 Neurology Unit, IRCCS San Raffaele Scientific Institute, Milan, Italy; 6 Center for Alzheimer’s disease and related disorders (CARD), IRCCS Ospedale San Raffaele, Milan, Italy; 7 Vita-Salute San Raffaele University, Milan, Italy; 8 Neuroimaging Research Unit, Division of Neuroscience, IRCCS San Raffaele Scientific Institute, Milan, Italy; 9 Neurotech Hub, Vita-Salute San Raffaele University, Milan, Italy; 10 Neurology Unit, Department of Medical, Surgical and Health Sciences, University of Trieste, Trieste, Italy; 11 Department of Medicine and Aging Sciences, ‘G. d’Annunzio’ University of Chieti-Pescara, Pescara, Italy; 12 Department of Human Neuroscience, Sapienza, University of Rome, Rome, Italy; 13 Scuola Universitaria di Studi Superiori IUSS di Pavia, Pavia, Italy; 14 IRCCS Istituto Auxologico, Milan, Italy; 15 Experimental Neuropsychophysiology, IRCCS Santa Lucia Foundation, Rome, Italy; 16 Department of Medicine and Surgery and Milan Center for Neuroscience ((NeuroMI), University of Milano-Bicocca, Milan, Italy; 17 Fondazione IRCCS San Gerardo dei Tintori, Monza, Italy; 18 Department of Biomedical, Surgical and Dental Sciences, University of Milan, Milan, Italy; 19 Center of Sleep Medicine, Department of Neurology, Villa Serena Hospital, Città Sant’Angelo, Pescara, Italy; 20 Department of Neuroscience, Psychology, Drug Research and Child Health, University of Florence, Florence, Italy; 21 IRCCS Fondazione Don Carlo Gnocchi, Florence, Italy; 22 Neuroscience Research Center, Department of Biomedical and Clinical Sciences, University of Milan, Milan, Italy; 23 Cognitive and Memory Disorders Clinic, AOUP “Paolo Giaccone” University Teaching Hospital. Department of Biomedicine, Neurosciences, and Advanced Diagnostics (Bi.N.D.), University of Palermo, Palermo, Italy; 24 Aging Brain and Memory Clinic, Department of Neuroscience, University of Torino, Torino, Italy; 25 Fondazione IRCCS Istituto Neurologico Carlo Besta, 20133 Milan, Italy; 26 Università degli Studi di Parma, Brunel University, London , UK; 27 Department of Neuroscience (DNS) and Padua Neuroscience Center (PNC), University of Padova, Padova, Italy; 28 Department of Neurosciences “Rita Levi Montalcini”, University of Turin, Neurology 2 Unit, A.O.U. Città della Salute e Della Scienza di Torino, Turin, Italy

**Keywords:** Delphi consensus, Italy, Dementia, Disease modifying drugs, Biological diagnosis, Alzheimer care management

## Abstract

**Abstract:**

This national expert-based Delphi-consensus aims at formulating recommendations on the management of dementia care in Italy. This effort seems important and timely given in light of a new scenario arising from a new biological definition of Alzheimer’s disease (AD) and the availability of disease-modifying treatments (DMTs).

**Methods:**

the Steering Committee of the Italian Neurological Society for dementia (SINdem) created appropriate statements. Invited SINdem experts were requested to vote on the statements according to a modified three-round Delphi method. Only those statements reaching Grade A (full agreement ≥ 75%) or B (overall agreement ≥ 80% and full disagreement < 5%) were included in the final document. Round answers’ consistency was graded using the Cohen’s k and within-class correlation coefficient.

**Results:**

Forty-six experts voted on 20 statements, which focused on the following aspects: i) organization of care services from early diagnosis to the management of advanced clinical stages; ii) access to biomarkers for a biological diagnosis of AD; iii) requirements necessary for the administration of DMTs; iv) main actors and pathways for the management of patients suffering from cognitive disorders. At the end of the process, 4 statements (20%) received a Grade A consensus, while 16 (80%) reached a Grade B consensus. Although the responses reflect heterogeneity among Italian regions, there was a fair degree of consistency for all statements.

**Conclusion:**

The high strength of this expert-based Delphi-consensus may offer guidance for improving the patient’s journey of individuals with cognitive decline from a biological diagnosis to DMTs administration and may possibly offer hints to the Health Systems on dementia.

**Supplementary Information:**

The online version contains supplementary material available at 10.1007/s10072-025-08143-5.

## Introduction

Dementia is a public health priority and a major cause of disability and dependency among older adults worldwide [1]. Alzheimer's disease (AD) accounts for 60–65% of cases. To better define prevention and therapeutic strategies, AD needs to be distinguished from other forms of neurodegeneration and vascular dementia, particularly in its early clinical stages. Nonetheless, the distinction between different forms of cognitive decline is often blurred, with mixed pathologies frequently coexisting in patients’ brain tissue [2], especially in older individuals. The consequences of dementia extend beyond financial implications, encompassing substantial costs for countries, societies, families, and of course, the patients themselves [3].

The significant clinical, epidemiological, and socio-economic challenges posed by dementia prompt the urgency of addressing this condition, as indicated by the Global Action Plan on the Public Health Response to Dementia 2017–2025 [3, 4].

In recent years, a significant change in the diagnostic pathway has occurred in the dementia field, due to the availability of in vivo AD biomarkers. These achievements have changed the diagnostic pathway of patients with cognitive decline from a clinical to a biological definition of the disease.

From a treatment point of view, a better understanding of the neuropathological processes typical of AD has paved the way for developing novel pharmacological interventions designed to modify the disease's trajectory [5–9]. Most of these drugs are primarily targeted to remove β-amyloid plaques [10–12] from the brain or block other pathophysiological events that follow β-amyloid plaque aggregation (i.e., tau phosphorylation, inflammation, neuronal redox stress) [13]. These pharmacological approaches would require highly specialized experts to diagnose dementia, highly specialized centers for disease-modifying treatments (DMTs) administration, and diffuse brain imaging availability to monitor adverse events.

In this scenario, it is crucial to be able to formulate an early biological diagnosis of AD. Identifying AD in its prodromal clinical stages is essential to enhance the potential efficacy of new biological therapeutic interventions. This requires the potentiation of infusion center in hospitals alongside an increased availability of MRI facilities and trained neuroradiologists.

A previous epidemiological study conducted in Italy, which focused on individuals older than 65 years, reported a prevalence of mild cognitive impairment (MCI) of 7.7% [14]. This implies that about 1,082,000 individuals in Italy are currently experiencing MCI. Considering that the prevalence of prodromal AD varies from 12% (individuals aged 50–54 years) to 24% (individuals aged 75–79) [15], we may anticipate that about 200,000 MCI individuals in Italy would require a biological diagnosis of AD, with a proportion of them being eligible for DMTs.

These figures make it clear that advances in DMTs availability and the need for a biological diagnosis of AD do not align with the current organization of the dementia centers in Italy, in which the management of dementias often relies only on a clinical definition of cognitive decline, as based on national plans and strategies [16].

The diagnosis and management of cognitive impairments and dementia in Italy are currently provided by more than 500 memory clinics, also known as Centers for Dementia and Cognitive Decline (CDCDs) [17]. These are specialized centers that offer multidisciplinary assessment, diagnosis, and treatment for patients with cognitive disorders, including those in the advanced stage of dementia. According to a 2023 survey conducted by the Italian Ministry of Health [18], Italian CDCDs are characterized by heterogeneous resources and regional inequalities in terms of diagnostic procedures (e.g., structural and functional neuroimaging, cerebrospinal fluid biomarkers for AD, etc.) and healthcare professionals involved in the services (i.e., neurologists or geriatricians, administrative staff, neuropsychologists, rehabilitation specialists, etc.). Up to 10% of centers reported not to be equipped for delivering a comprehensive neuropsychological assessment, while about 40% of centers reported their inability to arrange the investigations needed for a biological diagnosis of AD. Moreover, the availability and accessibility of memory clinics varied significantly depending on the geographical area of Italy, generally being lower in southern regions and in rural and remote areas of the country. More than half of the CDCDs were hospital-based (51% mainly distributed in northern regions), while 42% of them were territorially based (mainly distributed in Southern regions), and 7% were university centers, involving more than 1,500 healthcare professionals in total.

Against this background, the logistic organization of the CDCDs system appears not to be ready yet for implementing new diagnostic pathways for a biological definition of AD, and for the selection and monitoring of candidates to DMTs.

For this reason, the Italian Scientific Societies with a special interest in dementia are urged to play a role in identifying and promoting the most appropriate and cost-effective strategies to be implemented in clinical practice. The use of DMTs in AD involves collaborative efforts between academia, scientific societies, industry, patients and regulatory policies.

Within this context, the Italian Society of Neurology for the Study of Dementia (SINdem) implemented a Delphi consensus study among its experts, who were selected homogenously from North, Center and South of Italy. The principal aims of the current study were to identify critical issues related to patient access, resources’ affordability, regional disparities across Italy, and implementation of up-to-date clinical and biological approaches into clinical practice.

## Methods

Study design and selection of experts.

A modified Delphi consensus process [19] was used to obtain expert consensus on the management of dementia from early diagnosis to advanced stages, considering the current organization of services and continuity of care. The study was conceived and designed by SINdem (Italy) and the Hygiene Section of the Department of Life science and Public Health of the Università Cattolica del Sacro Cuore (UCSC) (Italy). The survey was submitted online and recorded through SurveyMonkey (https://www.surveymonkey.com) in three rounds from August to December 2023.

SINdem members representative of the local organization of the Society across every Italian region were considered eligible as experts and invited by email to take part in the study. All of them had to be clinicians with a special interest in dementia and proven experience in the clinical management of patients with cognitive decline. Upon acceptance, participants were first required to provide their informed consent. The entire survey took approximately 15 min to complete. Data were collected with a near-anonymization method and analyzed in aggregate form.

Delphi survey and methodology.

The SINdem Scientific Committee produced the survey statements, which were then adapted for a Delphi consensus by F.B. and G.E.C. from the Hygiene Section of the UCSC Department of Life science and Public Health.

Statements were divided into four themes, as clarified here.

Theme 1: The organization of care services from early diagnosis to the management of advanced stages;

Theme 2: Access to biological diagnosis of AD;

Theme 3: Requirements for the administration of Disease-Modifying Treatments (DMTs);

Theme 4: Taking charge, actors and management of patients with cognitive disorders.

A total of 20 statements were submitted for scoring as 4-point Likert questions, followed by 4 open questions (one at the end of each theme). In open-questions respondents were required to express their own thoughts on each subject. Seven additional questions were included to collect sociodemographic data and information on the work activities of all respondents.

### Statistical analysis

Statistical analysis was performed using STATA 18.0 software (Stata Corporation, College Station, TX, USA).

The statements were tested in a three-round Delphi process, using a 4-point Likert scale, where 1 corresponds to full agreement and 4 to full disagreement. At consensus, the statements were evaluated according to the strength of agreement and ranking consistency, calculated from the previous round. The methodology is summarized in Supplementary Table 1.

In addition to agreement, the mean score and standard deviation, and the significance of change from the previous round were evaluated using the Wilcoxon-Mann–Whitney test and Pearson's correlation. These findings were used to confirm the strength of consensus. A p-value < 0.25 was considered as indicative of a significant variation, considering that some degree of multiplicity was expected. Consistency was assessed based on intraclass correlation coefficients and p-values, Cohen's kappa, and Fleiss' pi and test–retest reliability by Bland–Altman plot. (Supplementary Table 1).

The proportion of ratings exceeding the critical difference was estimated for test–retest reliability according to Bland and Altman. A proportion of outliers above 10 percent was considered indicative of significant heterogeneity among experts and was used as cut-off for downgrading consistency.

The decision to refuse or modify and resubmit a statement was based on a composite of different statistical criteria. The pre-defined criteria for submission or resubmission after the first round were set as follows: statements with a proportion of full disagreement ≥ 10% and/or a mean score < 2.0 were not resubmitted. In all other cases, statements were resubmitted after textual adaptations and/or merging, as appropriate. Statements with a proportion of overall agreement < 80% and a proportion of full disagreement > 5% (Grades C and D) were removed from the consensus after the second round.

At the time of consensus, statements with grade A and B strength were included in the final set of recommendations.

## Results

Seventy-four SINdem experts were initially contacted and invited to participate in the consensus study. Fifty-eight of them replied to the first round (response rate 78.4%), and 46 replied to the second and third rounds (response rate 62.2%). Characteristics of the respondents are summarized in Table 1.

**Table 1:** Sociodemographic data and background description of experts

VARIABLES		ROUND	
		1	2 and 3
		N (%)	N (%)
*SEX*	Female	34 (58.62)	24 (52.17)
	Male	24 (41.38)	22 (47.83)
*REGION*	Northern Italy	32 (55.17)	20 (43.48)
	Centre Italy	14 (24.14)	12 (26.09)
	Southern Italy	7 (12.07)	7 (15.22)
	Islands	5 (8.62)	7 (15.22)
*AGE CLASS*	30–40	8 (13.79)	6 (13.04)
	41–50	12 (20.69)	8 (17.39)
	51–60	20 (34.48)	16 (34.78)
	61–70	18 (31.03)	16 (34.78)
*YEARS OF WORKING EXPERIENCE ON DEMENTIA*	0–5	2 (3.45)	2 (4.35)
	6–10	8 (13.79)	7 (15.22)
	11–15	13 (22.41)	4 (8.70)
	16–20	6 (10.34)	7 (15.22)
	> 20	29 (50.00)	26 (56.52)
*% CLINICAL PRACTICE DEDICATED TO PATIENTS WITH DEMENTIA*	0–20	11 (18.97)	5 (10.87)
	21–40	10 (17.24)	10 (21.74)
	41–60	18 (31.03)	17 (36.96)
	61–80	14 (24.14)	10 (21.74)
	81–100	5 (8.62)	4 (8.70)
*MEDICAL SPECIALTY*	Neurologist	56 (96.55)	43 (93.48)
	Geriatrician	2 (3.45)	3 (6.52)
*WORK CENTRE*	CDCD hospital	27 (46.55)	24 (52.17)
	CDCD territory	6 (10.34)	6 (13.04)
	CDCD university	25 (43.10)	16 (34.78)

The experts were mainly neurologists (93%), from Northern Italy (43%) and working in hospital-based CDCDs (52%). Fifty-seven percent of respondents had more than 20 years of working experience, and 37% dedicated nearly half of their working time to patients with dementia.

After the first round, two statements (13 and 19) were amended based on the criteria stated in the methods and according to the suggestions collected through the open-ended questions (Supplementary Table 2).

Table 2 summarizes the proportion of consensus obtained for each statement at the third round. At the end of the process, four statements (20%) received a Grade A consensus strength, and 16 statements (80%) reached a Grade B consensus strength.

**Table 2:** Strength of each statement in the Delphi consensus

Statement	Full agreement %	Overall agreement %	Full disagreement %	Mean	SD	Wilcoxon p value	Pearson p value	Strength grading
1	76.09	100	0	1.239	0.431	0.2500	< 0.0001	A
2	67.39	100	0	1.326	0.474	0.6875	< 0.0001	B
3	30.43	91.30	2.17	1.804	0.654	1.0000	< 0.0001	B
4	73.91	97.83	0	1.283	0.502	1.0000	< 0.0001	B
5	89.13	97.83	0	1.13	0.4	0.5000	< 0.0001	A
6	52.17	95.65	2.17	1.543	0.657	0.6133	0.0019	B
7	43.48	93.48	0	1.63	0.61	0.5078	< 0.0001	B
8	39.13	100	0	1.609	0.493	0.1562	0.0054	B
9	60.87	97.83	0	1.413	0.541	1.0000	< 0.0001	B
10	58.70	95.65	0	1.457	0.585	1.0000	< 0.0001	B
11	80.43	100	0	1.196	0.401	1.0000	0.0013	A
12	54.35	97.83	0	1.478	0.547	1.0000	< 0.0001	B
13	32.61	89.13	0	1.783	0.629	0.7949	1.0000	B
14	67.39	100	0	1.326	0.474	1.0000	< 0.0001	B
15	56.52	97.83	0	1.457	0.546	1.0000	< 0.0001	B
16	71.74	100	0	1.283	0.455	0.6875	0.0001	B
17	78.26	100	0	1.217	0.417	1.0000	0.0297	A
18	45.65	97.83	0	1.565	0.544	1.0000	< 0.0001	B
19	58.70	89.13	2.17	1.543	0.751	1.0000	< 0.0001	B
20	17.39	91.30	0	1.913	0.509	0.6250	< 0.0001	B

Table 3 summarizes the estimates of consistency across the second and third round. Most statements were classified as having fair consistency—Grade III (65%). These data suggest an acceptable but not completely homogeneous distribution of the values assigned to most of the statements by different experts.

**Table 3:** Estimates of consistency after three rounds in the Delphi consensus

Statement	Agreement %	Cohen’s Kappa		Fleiss Pi		Intraclass Correlation		Test–retest	Overall consistency
		**Coeff**	**P value**	**Coeff**	**P value**	**Coeff. (95%CI)**	**P value**		
1	0.935	0.802	< 0.001	0.801	< 0.001	0.0444 (−0.009 0.986)	0.083	6.52	II
2	0.870	0.713	< 0.001	0.712	< 0.001	–0.007 (−0.019 0.935)	0.420	13.04	III
3	0.848	0.723	< 0.001	0.722	< 0.001	–0.019 (−0.0216 0.752)	0.710	13.04	III
4	0.9348	0.820	< 0.001	0.819	< 0.001	0.0444 (−0.009 0.986)	0.083	6.52	II
5	0.9565	0.813	< 0.001	0.812	< 0.001	0.0222 (−0.0136 0.978)	0.160	4.35	II
6	0.739	0.526	< 0.001	0.526	< 0.001	–0.0089 (−0.0197 0.928)	0.445	26.09	III
7	0.804	0.644	< 0.001	0.644	< 0.001	3.52e-16 (−0.018 0.956)	0.323	19.57	III
8	0.870	0.749	< 0.001	0.748	< 0.001	0.0444 (−0.009 0.986)	0.083	8.70	II
9	0.891	0.777	< 0.001	0.777	< 0.001	–0.0178 (−0.0214 0.810)	0.660	10.87	III
10	0.826	0.664	< 0.001	0.664	< 0.001	–0.0222 (−0.0222 –0.0222)	1.000	17.39	III
11	0.891	0.639	< 0.001	0.639	< 0.001	−0.0178 (−0.0214 0.810)	0.660	10.87	III
12	0.913	0.833	< 0.001	0.833	< 0.001	–0.0222 (−0.0222 –.0222)	1.000	8.70	II
13	0.783	0.625	< 0.001	0.625	< 0.001	–0.007 (−0.0194 0.935)	0.420	19.57	III
14	0.956	0.901	< 0.001	0.901	< 0.001	–0.0222 (−0.0222 –0.0222)	1.000	4.35	II
15	0.891	0.787	< 0.001	0.787	< 0.001	–0.0178 (−0.0214 0.810)	0.660	10.87	III
16	0.870	0.693	< 0.001	0.692	< 0.001	–0.007 (−0.0194 0.935)	0.420	13.04	III
17	0.848	0.568	< 0.001	0.568	< 0.001	–0.019 (−0.0216 0.752)	0.710	15.22	III
18	0.870	0.749	< 0.001	0.749	< 0.001	–0.0222 (−0.0222 –0.0222)	1.000	13.04	III
19	0.891	0.805	< 0.001	0.805	< 0.001	–0.0178 (−0.0214 0.810)	0.660	8.70	II
20	0.913	0.792	< 0.001	0.792	< 0.001	–1.26e-15 (−0.0180 0.956)	0.323	23.91	III

In Table 4 are listed the final statements with the strength of the consensus and the consistency.

**Table 4:** Twenty final Grade A and B recommendations included after three rounds in the Delphi consensus

Statement number	Statement	Strength	Consistency
1	Each Region must have a Diagnostic, Therapeutic and Assistance Pathway (PDTA) for dementia in accordance with the indications of the National Dementia Plan	A	II
2	It is desirable that the General Practitioner (GP) applies risk maps to implement prevention policies and intercept suspected patients	B	III
3	In the suspicion of cognitive deficits, the GP must perform a screening test (e.g. GP-Cog) before selecting patients to be referred to the CDCD	B	III
4	It is necessary to integrate the CDCDs of the territory and those of the hospital in order to respond to different needs and phases of dementia according to their specificities	B	II
5	It is necessary to integrate the non-CDCD care phases into a network of integrated services (territorial specialists, day centers, social and health services), especially with regard to the advanced and or complex stages of the disease on the basis of what is described in the regional PDTAs	A	II
6	The current organization of the CDCD does not allow the biological diagnosis of Alzheimer's disease to be carried out uniformly throughout the country	B	III
7	In order to allow all patients to have access to biological diagnosis, the CDCD network, based on their different functions, must be organized at district level (hub and spoke model)	B	III
8	To obtain a biological diagnosis, CSF testing provides more clinically useful pathology information than amyloid PET	B	II
9	It is appropriate to request investigations for a biological diagnosis of AD in those people considered best candidates for DMTs, on the basis of the prescriptive restrictions provided by the authority in Countries where these treatments are licensed	B	III
10	The use of plasma biomarkers can be implemented as a preliminary investigation to optimize screenings aimed at selecting subjects to undergo more invasive or expensive investigations for the NHS	B	III
11	In order to proceed with the correct prescription, administration and monitoring of DMTs, it is necessary to reorganize and strengthen the day hospitals, radiology services with specific focus on the personnel training	A	III
12	The prescription of disease-modifying drugs must be under the responsibility of CDCDs	B	II
13	The administration of DMTs can also be carried out by specialized hospitals as long as they are able to guarantee safety in the administration and clinical-radiological monitoring	B	III
14	It is necessary that CDCDs, which are infusion centers for DMTs, are also able to perform clinical-radiological monitoring	B	II
15	CDCD identified as infusion Centers must have specific characteristics (possibility of access to Emergency Department H/24 h a day, facilitated contact with the reference specialist)	B	III
16	PDTAs are essential for the organization of health and social care for people with neurocognitive disorders and for proper management	B	III
17	Patient care cannot be linked only to the CDCD but must be organized at a local level involving GPs, neurology and territorial geriatricians, day centers, Alzheimer’s café networks, Community Hospitals and nursing homes	A	III
18	Patients/caregivers associations play an important supporting role in the social-welfare process	B	III
19	AIFA note 85 can be abolished by ensuring that patients are taken care of by CDCDs for the appropriate diagnostic care pathways	B	II
20	Telemedicine should be used in CDCDs for follow-up visits, follow-up cognitive assessments, for therapeutic adjustments, and for speech and cognitive rehabilitation/stimulation	B	III

Legend: *CDCD*: Centers for cognitive decline and dementia; *DMTs*: disease modifying treatments; *AIFA*: Italian Regulatory Drugs Agency

## Discussion

The statements proposed in this study provide an overview of the critical issues that define the necessary levels of organization for an efficient management of patients within the dementia services. This includes not only an accurate diagnosis of the disease, supported by a neurobiological evidence, but also a competent management of more advanced clinical stages.

In detail, experts recognized the adherence of Diagnostic, Therapeutic and Assistance Pathways (PDTAs) to the National Dementia Plan and the integration of care system as crucial. This assigns to the General Practitioner (GP) the critical role of “system gatekeeper” to ensure active collaboration between CDCDs and non-CDCD care facilities. Patients’ and caregivers’ associations are also regarded as playing a key role in the area of therapeutic alliance. In addition to cross-integration between different stakeholders, a hub-and-spoke model of organization is considered recommendable. In the light of new scientific evidence, particular attention is given to a biologically based diagnosis. Within the framework of biological diagnosis, an added value is recognized for CSF biomarkers over neuroimaging biomarkers. Regardless of the type of biomarkers chosen, a biological approach is considered essential to identify patients who are potentially eligible for DMTs.

In addition, attention is given to the necessary facilities that qualify CDCDs as centers capable of offering DMTs to eligible patients. DMTs infusion prescription and administration require an adequate setting in terms of quality and safety of care, including, for instance, facilities for radiological monitoring. Prescriptive and organizational innovations that challenge the policies currently in place within the Italian NHS are also mentioned in the last statements. Substantial agreement was achieved about the abolition of the AIFA (Agenzia Italiana del Farmaco; i.e. Italian Agency for drug Regulation) Note 85 which imposes mandatory administrative procedures for the prescription of cholinesterase inhibitors and memantine. The abolition of Note 85 is not perceived as a factor that would reduce the CDCD's central role in the diagnostic process and management of people with dementia. The crucial role of telemedicine in this Delphi consensus is in line with the PNRR (i.e., National Recovery and Resilience Plan) reform plan on the digitization of healthcare. Nonetheless, experts recognized the substantial current immaturity of the NHS in adopting tools for the digitalization of services, with significant regional and local divergence in patient management. This issue is also reflected by the results of the current study, which show a low degree of consistency achieved across most of the statements. This is particularly evident when considering the non-significant p-values obtained for inter-rater agreement, although a very high consensus strength (Grade A and B) was still achieved.

The eagerly anticipated era of DMTs for AD patients is approaching, and this revolution requires a profound modification of the way the disease is regarded and managed, generating new questions and concerns [5, 20].

Italy has a universalistic health care system. This means for dementia patients that the NHS would assess the prescription and distribution of therapies within CDCDs, with accountability for skills and tasks through a multilevel organization with increasing complexity. Additionally, there is the need for CDCDs to be networked to ensure continuity of care through the integration of different professional figures, including GPs, geriatricians, neurologists, psychiatrists, neuroradiologists, psychologists, laboratory technicians, nuclear medicine physicians, nurses, therapists, and rehabilitation operators. A better definition of the criteria for accessibility to a biological diagnosis of cognitive decline is also needed, due to its consequences for the organization of services. For instance, lumbar puncture for CSF biomarker analysis is currently not incorporated into the Italian Essential Levels of Care (LEA). This means that this procedure is not reimbursed by the NHS in most areas of Italy (except for Umbria Region). Moreover, assessing the risk-to-benefit ratio of using anti-Aβ immunotherapies requires highly specialized diagnostic facilities, which are currently available only in high-complexity clinical settings. Management of amyloid-related imaging abnormalities (ARIA) requires standardized clinical and neuroradiological protocols in both routine and emergency clinical settings. Appropriate and dedicated neuroradiological centers need to be identified in terms of capacity and expertise, and additional centers need to be prepared to offer safe patient monitoring with newly trained operators.

Much like DMTs and thrombolysis have changed the management of patients with multiple sclerosis and stroke, emerging therapies have the potential to offer a similar revolutionary transformation in neurodegenerative dementia. This involves creating new treatment plans, establishing dedicated care pathways, and expanding multidisciplinary teams. In the past, these adjustments have helped patients achieve better outcomes and have accelerated the acceptance of novel therapies [5, 21]. A new organization of CDCDs, in which different minimum requirements are expected based on varying levels of expertise in the diagnostic pathway, has already been proposed in a previous work [15]. However, this earlier paper does not consider the whole patient journey, from the GP’s case finding to the full management of patients and caregivers.

The present study aims to pave the way for a different diagnostic pathway for the biological diagnosis of dementia, with the goal of preparing health services for a new model of care and management of patients, especially those with AD. Through an examination of the most critical issues highlighted by current clinical practice, and considering national strategies and regional and local PDTAs, the recommendations provided by SINdem may serve as fertile ground for future national and regional strategies and guidelines.

This study has some limitations. First, it reflects the viewpoint of SINdem members, whose composition mainly involves neurologists. For this reason, some issues regarding the complex scenario of dementia care might not have been comprehensively addressed. Similarly, it is possible that some relevant topics have not been adequately covered by the statements included in the current study. However, the open-ended questions and the rigorous methodology employed here were designed to mitigate these limitations to some extent. It should be noted that all experts involved in the study have extensive clinical experience in the area of dementia and have been working in CDCDs located in different areas of the Country.

Given the range of the population affected directly or indirectly by dementia and the complexity of this condition, dementia requires a whole-of-government, broad, multistakeholder, public health approach. Such an approach will lead to a comprehensive response from the health and social care system (both public and private) and other government sectors, and will engage people with dementia, their carers, and other relevant stakeholders and partners [4].

## Conclusion

The new scenario of changes around the management of patients with cognitive decline poses challenges to health systems, healthcare workers, and patients. An integrated pathway and clear-cut management of patients are needed and expected to be beneficial in reducing social and economic costs. SINdem recommendations offer guidance for policy makers, highlighting bottlenecks and suggesting cost-effective improvements. These recommendations aim at addressing the heterogeneity of dementia service organizations in Italy and call for timely action [16–18, 21].

Furthermore, several activities should be pursued to advance the value-based approach [22] in dementia care. In conclusion, enhancing evidence and data generation is crucial for shaping dementia policies based on new or improved assessment frameworks that fully appreciate the value of innovative drugs. The transition to a value-based dementia management approach should also rely on the active and well-informed participation of all pertinent stakeholders. Finally, considering some similarities between the Italian NHS and those of other Countries, the present work might contribute to a wider synergistic effort for the standardization of quality care in dementia.

## Supplementary Information

Below is the link to the electronic supplementary material.

Supplementary file1 (DOCX 17 KB)

## Data Availability

On request to the corresponding Author.
