# Supplementary material for: Towards a new Value-based scenario for the management of dementia in Italy: a SINdem delphi consensus study
Source: Neurol Sci. 2025 Apr 16;46(7):2913–23. doi: 10.1007/s10072-025-08143-5 (PMC12152079; doi:10.1007/s10072-025-08143-5)
Supplement: Supplementary file 1 — Supplementary file1 (DOCX 17 KB) [file 10072_2025_8143_MOESM1_ESM.docx]

*Supplementary Table 1 Strength and consistency grading definitions for statement submitted to the expert panel during the Delphi rounds.*

| **Grade** | **Rating** | **Definition** |
| --- | --- | --- |
| A | Very strong | Full agreement ≥75% |
| B | Strong | Full agreement <75%  Overall agreement ≥80%  Full disagreement <5% |
| C | Fair | Full agreement <75%  Overall agreement ≥80%  Full disagreement ≥5% |
| D | Poor | Full disagreement ≥10% |
| **Consistency** | **Rating** | **Definition** |
| I | Very high | Cohen’s k and intraclass correlation coefficient, p value ≤0.001 in both analysis |
| II | High | Cohen’s k and intraclass correlation coefficient, p value  ≤0.001 in one and ≤0.010 in the other analysis |
| III | Fair | Fleiss’s k p value <0.0001 |
| IV | Poor | Fleiss’s k p value >.01 |

*Supplementary Table 2 Description of changes made to statements between rounds*

| **Statement number** | **1 round version** | **2 round version** |
| --- | --- | --- |
| 13 | The administration of DM drugs can also be carried out in local structures for chronicity (community hospitals) in a network with hospital CDCDs (for monitoring) in order to increase ease of access to treatments. | The administration of DM drugs can also be carried out by specialized hospitals as long as they are able to guarantee safety in the administration and clinical-radiological monitoring. |
| 19 | AIFA note 85 must be deleted | AIFA note 85 can be abolished by ensuring that patients are taken care of by CDCDs for the appropriate diagnostic care pathways |
